# Constant Light in Critical Postnatal Days Affects Circadian Rhythms in Locomotion and Gene Expression in the Suprachiasmatic Nucleus, Retina, and Pineal Gland Later in Life

**DOI:** 10.3390/biomedicines8120579

**Published:** 2020-12-07

**Authors:** Aneta Kubištová, Veronika Spišská, Lucie Petrželková, Leona Hrubcová, Simona Moravcová, Lenka Maierová, Zdeňka Bendová

**Affiliations:** 1 Department of Physiology, Faculty of Science, Charles University, 128 43 Prague, Czech Republic; 2 Department of Sleep Medicine and Chronobiology, National Institute of Mental Health, 250 67 Klecany, Czech Republic; anakubistova@seznam.cz (A.K.); veronika.spisska@natur.cuni.cz (V.S.); lucie.petrzelkova@gmail.com (L.P.); lea.hrubcova@gmail.com (L.H.); simona.moravcova@natur.cuni.cz (S.M.); 3 University Center for Energy Efficient Buildings, Czech Technical University in Prague, 273 43 Buštěhrad, Czech Republic; lenka.maierova@fsv.cvut.cz

**Keywords:** circadian clock, light at night, rat, suprachiasmatic nucleus, retina, pineal gland

## Abstract

The circadian clock regulates bodily rhythms by time cues that result from the integration of genetically encoded endogenous rhythms with external cycles, most potently with the light/dark cycle. Chronic exposure to constant light in adulthood disrupts circadian system function and can induce behavioral and physiological arrhythmicity with potential clinical consequences. Since the developing nervous system is particularly vulnerable to experiences during the critical period, we hypothesized that early-life circadian disruption would negatively impact the development of the circadian clock and its adult function. Newborn rats were subjected to a constant light of 16 lux from the day of birth through until postnatal day 20, and then they were housed in conditions of L12 h (16 lux): D12 h (darkness). The circadian period was measured by locomotor activity rhythm at postnatal day 60, and the rhythmic expressions of clock genes and tissue-specific genes were detected in the suprachiasmatic nuclei, retinas, and pineal glands at postnatal days 30 and 90. Our data show that early postnatal exposure to constant light leads to a prolonged endogenous period of locomotor activity rhythm and affects the rhythmic gene expression in all studied brain structures later in life.

## 1. Introduction

The light/dark cycle exerts a range of physiological responses in organisms, predominantly via synchronizing the circadian system. The mammalian circadian system is composed of a master circadian pacemaker in the suprachiasmatic nucleus (SCN) of the hypothalamus and peripheral oscillators in most tissues and organs. The SCN generates self-sustained rhythms in gene expression and electrical activity that oscillate with a circadian period of approximately 24 h and produce the humoral or electrical timing signals that are distributed throughout the body [1]. The molecular clockwork underlying this endogenous periodicity is based on the transcriptional/translational feedback loops of clock genes, such as *Period 1, 2, Clock, Bmal1, Cryptochromes, Nr1d1* (encoding nuclear receptor *Rev-erbα*), and *Rorα* [2].

Although the circadian system generates circadian rhythms even in complete darkness, its precise 24 h periodicity requires perpetual resetting by the solar light/dark cycle. Light information for the SCN is formed by the intrinsic photosensitive retinal ganglion cells (ipRGCs) containing the photopigment melanopsin (*Opn4*), which has maximum sensitivity within short-wavelength light (peak absorption ~479 nm) [3]. The ipRGCs combine rod- and cone- with melanopsin-driven activities and transduce them into the glutamatergic signal that affects the SCN molecular clockwork predominantly via NMDA receptors [4]. In addition to melanopsin, another non-visual opsin, *Opn5* (neuropsin), has been identified in the ganglion cell layer of rat retinas [5], which respond to UV light (peak absorption ~380 nm). It has been reported to drive the entrainment of retinal circadian oscillators to light/dark cycles independent of rods, cones, and *Opn4* [6].

A prominent endocrine manifestation of the circadian clock is the daily cycle of the melatonin level. Melatonin is synthesized by the pineal gland at night and in darkness, upon signaling from the SCN, and its rhythmic release to the bloodstream serves as a timing signal to peripheral oscillators. Melatonin biosynthesis in animals includes four enzymatic steps: first tryptophan hydroxylase (TPH) hydroxylates tryptophan to 5-hydroxytryptophan, which is then decarboxylated by aromatic amino acid decarboxylase to form serotonin. The rate-limiting enzyme, arylalkylamine-N-acetyltransferase (AANAT), plays a key role in the conversion of serotonin into N-acetylserotonin, and the last enzyme, N-acetylserotonin O-methyltransferase, catalyzes N-acetylserotonin to produce melatonin [7]. Inducible cAMP early repressor (ICER) is induced at night by noradrenaline in the pineal gland, and it may participate in regulating the decline in *Aanat* transcriptional rhythm toward the end of the night [8,9,10,11].

Although the alignment of the endogenous circadian with the exogenous solar period is generally called “light entrainment”, the high contrast between day and night intensity and the spectral change around dawn and dusk are necessary for the proper entrainment of the clock in nocturnal and diurnal animals [12,13]. Continuous exposure to light (LL) or even dim light at night (LAN) causes the desynchronization of SCN neurons and subsequently of the peripheral clock in bodily tissues [1,14,15,16,17] (also see [18]). The disruption of temporal coordination throughout the body has been associated with an increased risk of cancer, immune deficiencies, metabolic syndrome, type 2 diabetes mellitus, cardiovascular disease, psychiatric diseases, and sleep diseases in human and laboratory animal models [19].

Most up-to-date data present severe indications that LL or LAN can disrupt circadian physiology and the subsequent regulatory processes in the body in adulthood, but the evidence on its disruptive effect in early postnatal development on neuronal and physiological maturation is less clear. During ontogeny, the SCN maturates gradually. The spontaneous expression of the clock genes is detectable already before birth [20,21], and the amplitude of their rhythms increases during the first two weeks of life [22,23,24]. The neonatal rat SCN responds to acute light even before eye opening, but the entrainment of its circadian phase occurs only during the first two postnatal weeks [22].

Using the locomotor activity rhythm as a behavioral correlate of circadian clock function, the critical developmental period for the effect of LL on the maturation of the circadian system has been defined for rats between postnatal day 0 (P0) and P21 [25,26,27]. Since then, several studies have suggested that the LL condition during this lactation period has long-lasting effects on circadian system maturation: this affects the strength of the circadian pacemaker and its sensitivity to light in adulthood [28,29], impairs the circadian regulation of the metabolism [30], affects the morphology of the SCN [31,32] and SCN-targeted brain structures [32,33], and decreases day/night differences in plasma corticosterone and melatonin plasma levels [34,35].

Using a similar protocol, we tested the effect of development in LL during the lactation period on locomotor activity and gene expression in three principal brain parts of the circadian system: the SCN, retina, and pineal gland. It has been shown that LL rearing had a significant damaging effect on the retinas of albino mice and rats [29,36]. Although we worked with light substantially lower than the damaging intensity, we used pigmented Long–Evans rats, which allowed a better association of the obtained laboratory data with more natural ecological or biomedical observations.

Our data show that early postnatal exposure to LL leads to a prolonged endogenous period of activity and affects rhythmic gene expression later in life. The most affected structure was the pineal gland, where the majority of tested genes showed lowered amplitudes in animals reared in LL, followed by the SCN. In the retina, *Opn4, Opn5*, and rhodopsin (*Rho*), but not the gene for short-wavelength-sensitive (S) cone (*Opn1sw*), showed altered expression profiles in adulthood in animals raised under LL.

## 2. Materials and Methods

### 2.1. Animals and Experimental Design

Twenty female and 10 male Long–Evans rats (Charles River Laboratories International, Inc., delivered by Velaz, Ltd., Prague, Czech Republic) were maintained under a 12/12 h light/dark (LD) regime at a temperature of 23 ± 2 °C with free access to food and water at least 2 weeks before mating. The light source used in the study was a customized linear daylight white LED source (CCT 5630 K, Spectrasol, Prague, Czech Republic) with a dominant wavelength at 459 nm, producing uniform ground irradiance of 65.9 ± 3.0 mW/m^2^ (mean + SEM), that is, photopic illuminance 16.6 ± 1 lx (measured on the cage position). The mean photon flux was 1.91 × 1013 photons/cm^2^/s, with a mean melanopic irradiance of 20.6 ± 1 mW/m^2^. Irradiance and spectral characteristics were measured using a calibrated radiospectrometer (GL Spectis 1.0 Touch, GL Optics, Puszczykowo, Poland).

One day before the expected delivery, the light was switched on, and litters with dams were left undisturbed in constant light conditions (LL; 16 lux) until postnatal day 20 (P20). On P21, animals were returned to LD conditions (12 h of 16 lux/12 h of complete darkness) and left undisturbed until P30 or P90. The control animals were left on the LD regime throughout life.

On the day of the experiment, at P30, 72 control and 72 LL-exposed animals (4 females and 4 males at each time point) were released into constant darkness (DD) and sacrificed by rapid decapitation under sodium pentobarbital anesthesia in 3 h intervals, and the remaining animals were weaned from their mothers. On P60, the locomotor activity of 12 animals from each group were measured for 12 days in DD, and then all animals were returned to LD conditions. On P90, 54 animals (11 males and 15 females in the control group and 11 males and 17 females in the LL group) were sacrificed by rapid decapitation under sodium pentobarbital anesthesia in one-day DD conditions at 4 time points. Time was expressed as circadian time (CT), where CT0 corresponds to the time with lights on, and CT12 with lights off.

This study was carried out in strict accordance with the recommendations in the Guide for the Care and Use of Laboratory Animals of the National Institutes of Health. The protocol was approved by the Animal Protection Law of the Czech Republic (protocol number: MSMT-31592/2019-4, 2 December 2019).

### 2.2. Locomotor Activity Measurement

The locomotor activity of 11 control and 11 LL-exposed male rats at P60 was monitored in DD via infrared motion detectors (Mini-Mitter VitalView data-acquisition system). The circadian parameters of locomotor activity were detected using ClockLab analysis (version 6, Actimetrics, Wilmette, IL, USA). A free-running period was determined by using a Χ^2^ periodogram with 3 min resolution between 10 and 36 hours for 12 days in DD. The relative amplitude (RA) of the free-running rhythm was determined from a fast Fourier transformation. Nonparametric interdaily stability (IS) values are expressed in arbitrary units, ranging from zero for Gaussian noise to 1 for maximal IS, and decreasing with a higher day-to-day variation of the activity pattern [37].

### 2.3. Quantitative Real-Time RT–PCR

Pineal glands and retinas were immediately dissected and homogenized in RNAzol RT (Molecular Research Center). Total RNA was extracted using Direct-zolTM RNA MiniPrep (Zymo Research), in accordance with the manufacturer’s instructions. Total RNA (1 µg) was converted to cDNA by using the one-step SuperScriptTM VILOTM cDNA Synthesis Kit (Invitrogen, Carlsbad, CA, USA) according to the manufacturer’s instructions. The blocks of tissue containing the hypothalamus were sectioned into a series of 20 μm-thick coronal slices throughout the rostral-caudal extent of the SCN. The slices were stained with ethanolic cresyl violet for 60 s, and SCN regions were isolated using laser microdissection (LMD 6000; Leica Microsystems, Wetzlar, Germany) and immediately homogenized in RLT buffer (RNeasy Plus Micro kit; Qiagen, Hilden, Germany). Total RNA was extracted with the Rneasy Plus Micro Kit (Qiagen) according to the manufacturer’s instructions. We converted 1 μg of total RNA to cDNA using 2-step Enhanced Avian Reverse Transcriptase eAMV RT (Sigma-Aldrich, St. Louis, MO, USA) according to the manufacturer’s instructions. TaqMan^®^ PreAmp Master Mix (Life Technologies, Carlsbad, CA, USA) was used to pre-amplify small amounts of cDNA after reverse transcription.

Samples of cDNA (1 μL) were amplified in 20 μL of PCR reaction mixture containing 5× HOT FIREPol Probe qPCR Mix Plus (Baria, Prague, Czech Republic) and TaqMan probes (Life Technologies; Table 1). All qPCR reactions were performed in triplicate in a LightCycler 480 Instrument (Roche Life Science, Indianapolis, IN, USA), as described before [36]. The mean of the crossing point (Cp) obtained from qPCR was normalized to the level of the housekeeping gene *Gapdh* (retina and SCN) and *Hprt* (pineal glands) and used for the analysis of relative gene expression by the ΔΔCT method [38]. Due to a large number of samples, always two samples per time point were processed at once with a new set of housekeeping genes. Thus, the data are plotted as means of 6 to 8 values obtained from 4 ΔΔCT calculations.

### 2.4. Data Analysis and Statistical Procedures

The rhythmicity of gene expression was evaluated using one-way ANOVA for the effect of time and was further analyzed using cosinor analysis, as defined by the equation [Y = mesor + (amplitude* − cos(2*p*(X − acrophase)/wavelength], with a constant wavelength of 24 h, a horizontal line fit model (H0), and cosine curve as the alternative hypothesis. Rhythmicity was proved when both tests showed significant results. When rhythmicity was confirmed by both tests, the cosine curve parameters were calculated: mesor (the mid-value of the cosine curve representing a rhythm-adjusted mean), amplitude (the difference between the peak or trough and the mean value of the cosine curve), and acrophase (the time of the peak of fitted curve, representing the average time of high values in the data), which were compared by one-way ANOVA followed by Tukey’s multiple comparisons test. Two-way ANOVA was used with a post hoc pairwise comparison by the Sidak–Bonferroni method to compare the control and LL-exposed groups. Data are reported as the mean ± SEM of 6 to 8 animals. Two-tailed *t*-tests were performed to compare the trends in period length, amplitude, and interdaily stability of rhythm in a locomotor activity. The F test to compare the variances between groups was also considered. *p* < 0.05 was required for significance. All tests were performed in Graph Pad Prism 6 (GraphPad Software Inc., San Diego, CA, USA).

## 3. Results

### 3.1. Effects of Postnatal LL Condition on Locomotor Activity of Adult Male Rats

**Figure 1:**
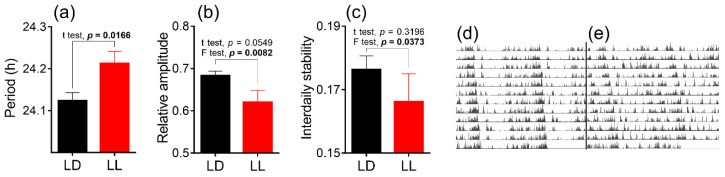
Effect of development under constant light (LL, in red; controls in light/dark (LD) in black) on (**a**) the circadian period, (**b**) relative amplitude, and (**c**) interdaily stability of the rhythm in the locomotor activity of male rats. Animals were housed under LL (16 lux) from the postnatal day 0 (P0) until P20 and then released into LD conditions. At P60, the animals were released into constant darkness (DD) and circadian parameters were calculated from 12 days of locomotor recordings. Each column represents the mean of 11 values ± SEM. *p*=; two-tailed Student’s *t*-test and F test comparing variances between groups; (**d**,**e**) representative actograms of a control animal and LL-exposed animal, respectively.

Locomotor activity measurement at P60 showed a significantly longer endogenous period in LL-reared animals compared to the light/dark (LD) group (Figure 1a). We also calculated relative amplitude (RA; Figure 1b) and interdaily stability (IS; Figure 1c). RA values reflect the differences in activity levels between the most and least active periods of the day, ranging from 0 (low variability) to 1 (clear distinction). IS represents the stability of the rhythm from one day to the next. A lower IS value means higher inter-daily variation. Although the means of RA and IS factors do not show a significant difference between groups, the data show a significantly different factor of variances, suggesting less stable rhythmicity in the LL-reared group. The total daily activities were similar between the groups (mean LD group = 3073.19 ± 123, mean LL group = 3092.41 ± 145).

### 3.2. Effects of Postnatal LL Condition on Gene Expression in the SCN

**Figure 2:**
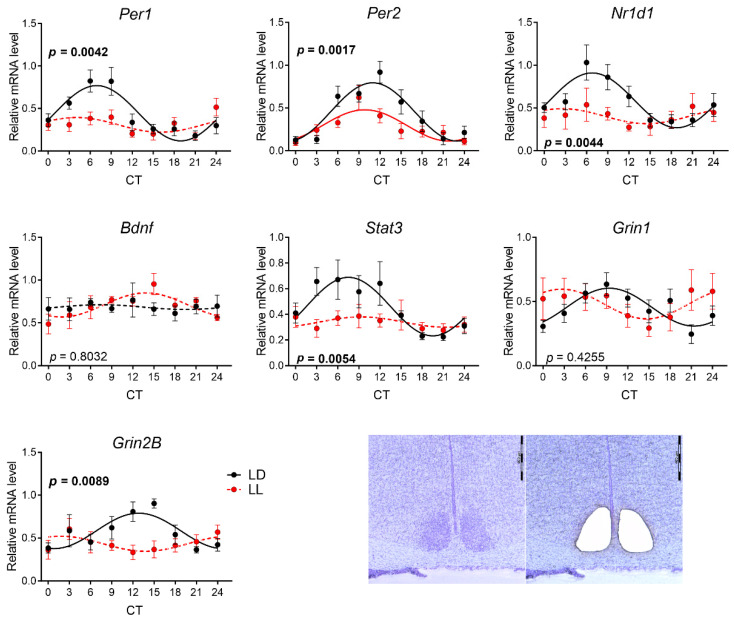
Effect of development under constant light (LL, in red; controls in LD in black) on the rhythmic gene expression in suprachiasmatic nucleus (SCN) at P30. Animals were housed under LL (16 lux) from P0 until P20 and then released into LD conditions. At P30, the animals were sacrificed in 3 h intervals in DD, and the SCNs were sampled by laser dissection and processed by qPCR with specific TaqMan probes. Rhythmicity was proved by cosinor analysis with the horizontal line as zero hypothesis and one-way ANOVA for differences between time groups. Full lines indicate rhythms confirmed by cosinor analysis and one-way ANOVA. Dashed lines indicate profiles not confirmed for rhythmicity by one or both methods. Each point represents the mean ± SEM from six to eight animals. *p*-values indicate group differences by two-way ANOVA, and significant values are in bold. Representative photomicrographs of coronal sections of SCN (right bottom) demonstrate an area of laser dissection. Scale bar = 400 μm.

To determine whether the LL condition during lactation affects the circadian rhythmicity of gene expression in the SCN, we performed qPCR on the SCN samples obtained by laser dissection (Figure 2). First, we evaluated the rhythms in P30 rats that were exposed to LL from P0 until P20, and then they experienced LD conditions for the next 9 days. The rhythmicity was proven when both one-way ANOVA (Supplemental Table S1) and the cosinor fit model analysis (Graph Pad Prism 6; Supplemental Table S2) showed significant results (see Materials and Methods). The analysis of our data confirmed the rhythmicity of all genes in the control LD group, with the exception of brain-derived neurotrophic factor (*Bdnf*) expression (Figure 2). In LL-reared animals, the rhythmicity of gene expression in the SCN was lost, with the exception of the *Per2* gene, whose rhythm in the LL group displayed a significantly lower mesor (*p* = 0.0005) and amplitude (*p* = 0.0091) compared to the control LD group (Table 2). The two-way ANOVA comparison revealed that the postnatal LL condition changed the rhythmicity in the SCN in all genes except NMDA receptor subunit *Grin1* (Figure 2, Supplemental Table S3).

**Figure 3:**
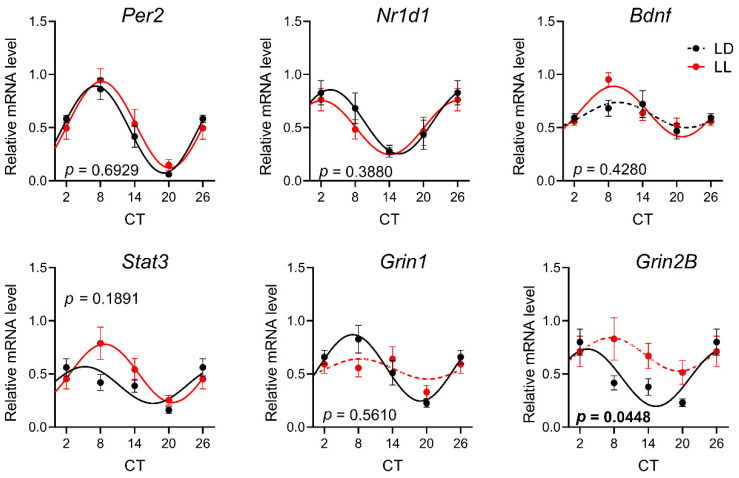
Effect of development under constant light (LL, in red; controls in LD in black) on rhythmic gene expression in SCN at P90. Animals were housed under LL (16 lux) from P0 until P20 and then released into LD conditions. At P90, the animals were sacrificed in 6 h intervals in DD, and SCNs were sampled by laser dissection and processed by qPCR with specific TaqMan probes. Rhythmicity was proved by cosinor analysis with a horizontal line as zero hypothesis and one-way ANOVA for differences between the time groups. Full lines indicate rhythms confirmed by cosinor analysis and one-way ANOVA. Dashed lines indicate profiles not confirmed for rhythmicity by one or both methods. Each point represents the mean ± SEM from five to seven animals. *p*-values indicate group difference by two-way ANOVA, and significant values are in bold.

The rhythmicity of gene expression in the SCN was evaluated at P90, when the animals experienced more than two months of the changing light/dark regime. Similar to the P30 control group, one-way ANOVA and the cosine fit model concurrently verify the significant rhythmicity, except for *Bdnf* expression. In LL-exposed animals, the arrhythmicity was confirmed for NMDA receptor subunits *Grin1* and *Grin2B* (Figure 3, Supplemental Tables S1 and S2). The two-way ANOVA comparison confirmed the different expression of the *Grin2B* gene (Supplemental Table S3), and the comparison of cosinor parameters revealed the different acrophase in *Stat3* expression (Table 2).

### 3.3. Effects of Postnatal LL Condition on Gene Expression in the Retina

**Figure 4:**
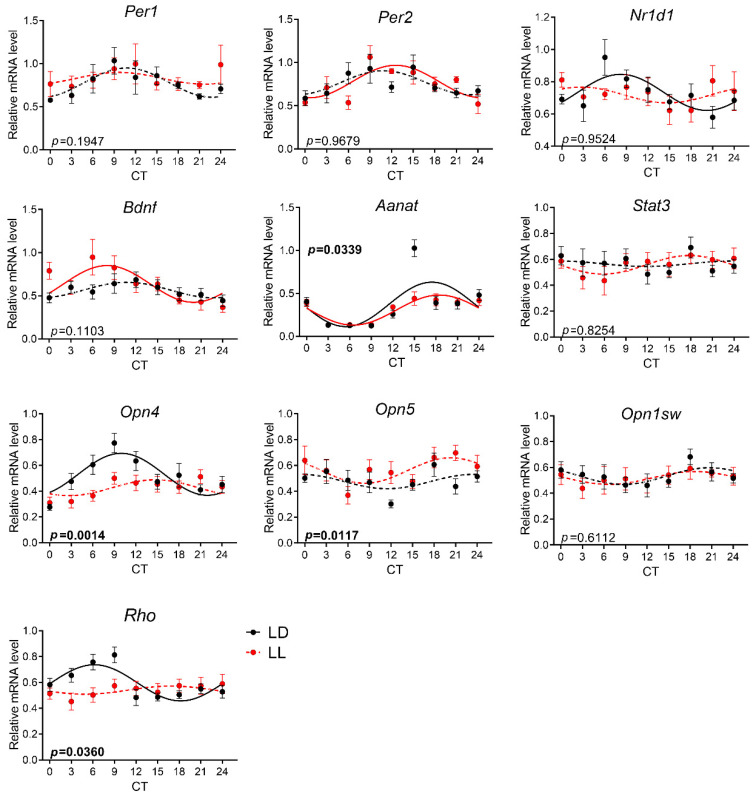
Effect of development under constant light (LL, in red; controls in LD in black) on rhythmic gene expression in retina at P30. Animals were housed under LL (16 lux) from P0 until P20 and then released into LD conditions. At P30, the animals were sacrificed in 3 h intervals in DD, and retinas were processed by qPCR with specific TaqMan probes. Rhythmicity was proved by cosinor analysis with a horizontal line as a zero hypothesis and one-way ANOVA for differences between the time groups. Full lines indicate rhythms confirmed by cosinor analysis and one-way ANOVA. Dashed lines indicate profiles not confirmed for rhythmicity by one or both methods. Each point represents the mean ± SEM from six to eight animals. *p*-values indicate group differences by two-way ANOVA, and significant values are in bold.

In contrast to the SCN, principal clock genes *Per1* and *Per2*, as well as *Stat3*, were not rhythmic in the control LD group of rats at P30 (Figure 4, Supplemental Tables S1 and S2). Similarly, *Opn5* and *Opn1sw* did not show circadian rhythmicity in LD or LL animals. *Nr1d1*, *Opn4*, and *Rho* had rhythmic expression in LD animals but lost rhythmicity in LL-reared animals. On the contrary, *Bdnf* expression was rhythmic only in the LL group. The two-way ANOVA comparison revealed that a postnatal LL condition changed the rhythmicity in the retina in *Aanat* and opsins *Opn4, Opn5*, and *Rho* (Figure 4 and Supplemental Table S3). Although the two-way ANOVA confirmed the difference between groups for *Aanat* expression, cosinor analysis did not confirm the difference for mesor or for amplitude or acrophase between the two rhythms (Table 2).

**Figure 5:**
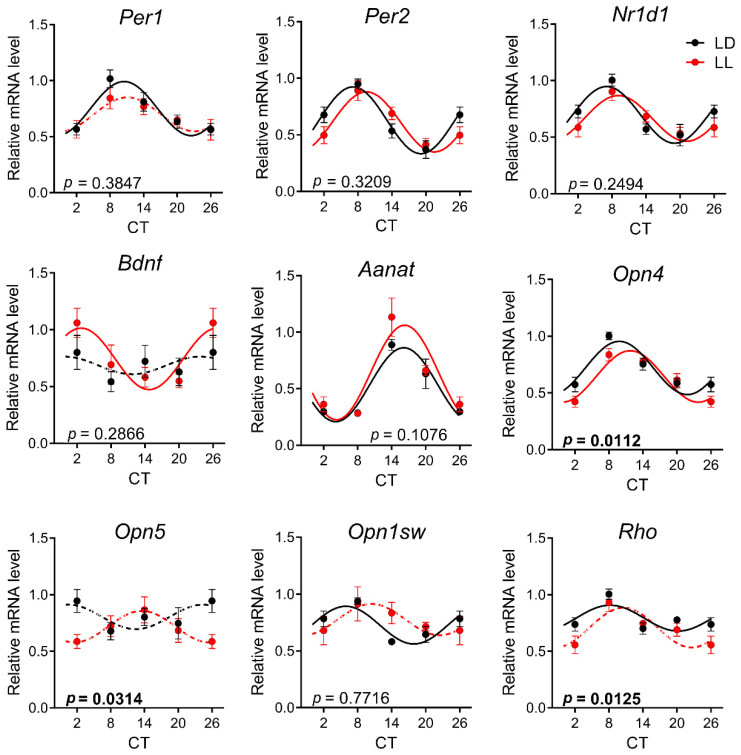
Effect of development under constant light (LL, in red; controls in LD in black) on rhythmic gene expression in the retina at P90. Animals were housed under LL (16 lux) from P0 until P20 and then released into LD conditions. At P90, the animals were sacrificed in 6 h intervals in DD and retinas were processed by qPCR with specific TaqMan probes. Rhythmicity was proved by cosinor analysis with a horizontal line as zero hypothesis and one-way ANOVA for differences between the time groups. Full lines indicate rhythms confirmed by cosinor analysis and one-way ANOVA. Dashed lines indicate profiles not confirmed for rhythmicity by one or both methods. Each point represents the mean ± SEM from five to seven animals. *p*-values indicate group differences by two-way ANOVA, and significant values are in bold.

At P90, the clock genes *Aanat, Opn4, Opn1sw*, and *Rho* showed circadian rhythmicity in control animals. LL rearing disrupted rhythms in *Per1, Opn5, Opn1sw*, and *Rho*, but similarly in P30, it induced rhythmicity in *Bdnf* expression (Figure 5, Supplemental Tables S1 and S2). The two-way ANOVA comparison confirmed the different expression for *Opn4, Opn5*, and *Rho* genes (Supplemental Table S3). A comparison of cosinor parameters (Table 2) between rhythms of *Opn4* expression revealed significant differences in mesor (*p* = 0.0390) and acrophase (*p* = 0.0267).

### 3.4. Effects of Postnatal LL Condition on Gene Expression in the Pineal Gland

**Figure 6:**
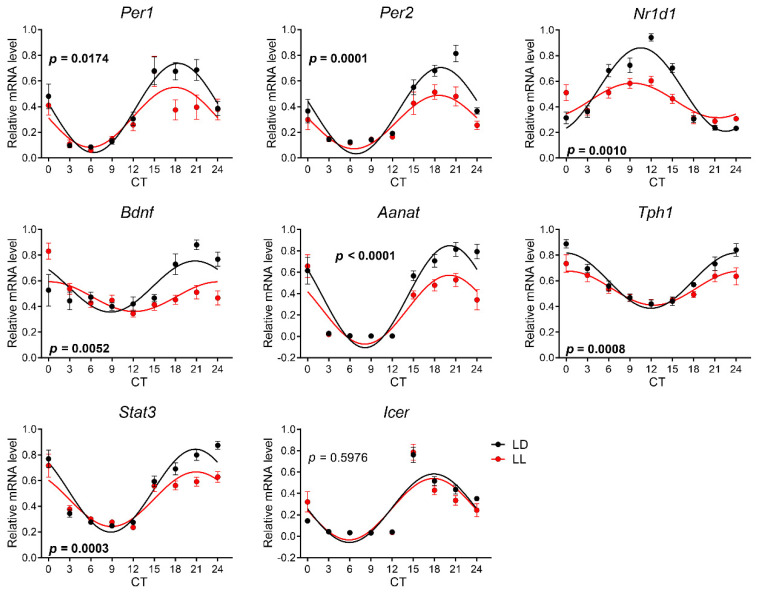
Effect of development under constant light (LL, in red; controls in LD in black) on rhythmic gene expression in the pineal gland at P30. Animals were housed under LL (16 lux) from P0 until P20 and then released into LD conditions. At P30, the animals were sacrificed in 3 h intervals in DD, and pineal glands were processed by qPCR with specific TaqMan probes. Rhythmicity was proved by cosinor analysis with the horizontal line as zero hypothesis and one-way ANOVA for the differences between time groups. Full lines indicate the rhythms confirmed by cosinor analysis and one-way ANOVA. Each point represents the mean ± SEM from six to eight animals. *p*-values indicate the group differences by two-way ANOVA, and significant values are in bold.

In the pineal gland, all genes were expressed rhythmically in LL-reared and LD-reared groups of rats at P30 (Figure 6, Supplemental Tables S1 and S2). All rhythms except Icer were significantly different between both groups, as confirmed by two-way ANOVA (Supplemental Table S3). A comparison of the cosinor parameters between rhythms revealed significant differences in the mesor and amplitude in all tested genes, except *Icer*, and phase-delayed acrophase in *Bdnf* expression in the LL group (Table 2).

**Figure 7:**
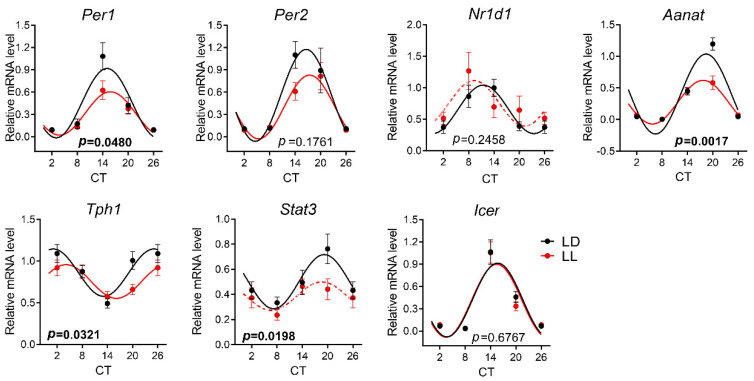
The effect of development under constant light (LL in red, controls on LD in black) on rhythmic gene expression in the pineal gland at P90. The animals were housed under LL (16 lux) from P0 until P20 and then released into LD conditions. At P90, the animals were sacrificed in 6 h intervals in DD, and pineal glands were processed by qPCR with specific TaqMan probes. The rhythmicity was proved by cosinor analysis with the horizontal line as a zero hypothesis and one-way ANOVA for the difference between time groups. Full lines indicate the rhythms that were confirmed by cosinor analysis and one-way ANOVA. The dashed lines indicate the profiles that were not confirmed for rhythmicity by one or both methods. Each point represents the mean ± SEM from five to seven animals. *p*-values indicate group difference by two-way ANOVA, and significant values are in bold.

Similar to P30, all expression profiles in the pineal glands of control animals at P90 showed circadian rhythmicity (Figure 7). LL rearing disrupted the circadian rhythm in *Nr1d1* and *Stat3* and significantly changed the waveform of the rhythm in *Per1*, *Aanat*, and *Tph1* (Supplemental Table S3). Table 2 demonstrates that the mesor values differ between rhythms in the expression of *Per1*, *Stat3*, and *Aanat*, and the amplitudes differ between the expression rhythms in *Per1* and *Aanat*.

**Figure 8:**
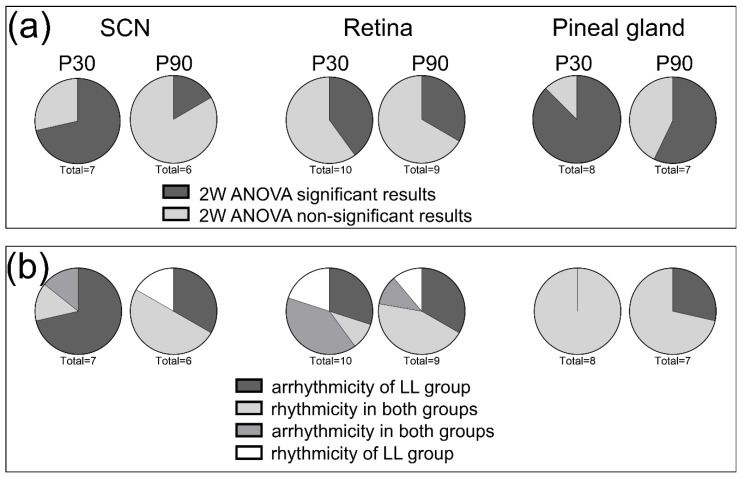
Pie charts showing (**a**) the relative distribution of statistically significant differences between LD and LL profiles of all gene expressions in brain structures obtained by two-way ANOVA; and (**b**) relative ratio of rhythmic and non-rhythmic expression profiles in both groups and all brain structures.

In general, all gene expression profiles were processed by three types of analysis. First, we compared the profiles between the LD and LL groups by 2W-ANOVA. Second, we assessed whether the LL conditions affected the result of combined 1W-ANOVA and cosinor analysis regarding the approval of circadian rhythmicity of each gene expression profile. Third, in case that gene expression was rhythmic in both groups, we tested whether prenatal LL conditions changed the cosinor parameters of the rhythm. Figure 8a depicts the relative ratio of significant and non-significant differences between the expression profiles obtained by two-way ANOVA, suggesting that the most affected structure was the pineal gland at P30. Figure 8b shows that the rhythmicity of all tested genes was preserved at P30 in the pineal gland, and only two genes became arrhythmic in the LL group at P90. Looking at the comparison of cosinor parameters in Table 2, however, we may notice that although all genes in the LL group show circadian rhythmicity, most of them differ from the LD group in mesor and amplitude. In the SCN, five of seven genes became arrhythmic in the LL group at P30, and Table 2 shows that *Per2*, which keeps the rhythmicity in both experimental groups, differs significantly in mesor and amplitude. The white part of the pie chart in Figure 8b indicates the rhythmicity that emerged only in the LL group. As mentioned above, this concerns the expression of *Bdnf* and, in the case of the retina at P30, also the *Per2* gene.

## 4. Discussion

The exposure of adult nocturnal rodents to LL disrupts the circadian rhythms of locomotor activity, body temperature, and plasma melatonin level; attenuates circadian rhythms in the cardiovascular system [16,39,40,41,42]; alters insulin secretion [41,43]; compromises the immune system [44]; and leads to anxiety and depression [45]. In this study, we determined the long-term effects of early postnatal LL conditions on the development of circadian oscillations in principal clock brain structures and activity rhythm. We adopted a model of exposure to LL conditions between P0 and P20. This interval has been determined as a critical period in which LL affects the development of rhythms in locomotor activity [25,26]. After P20, our rats were kept under LD cycles to explore whether recovery from unnatural lighting conditions would occur later in life with exposure to synchronizing lighting conditions. All animals were released into DD only on the day of sacrifice to unmask the acute effect of light on the circadian clock.

Compared to previous studies, we used a relatively low level of light, which was set based on the average measurement of LED streetlights. The intensity of 16 lux also does not present a risk for immediate retinal damage and diminishes the light-induced stress responses that could modify the results.

Our data showed that LL-reared pigmented Long–Evans rats exhibited longer endogenous periods compared to LD-reared animals when tested around P60. It has been demonstrated previously that rats maintained under LL during lactation expressed a circadian rhythm in locomotor activity when placed into DD conditions later in life [46]. Changes in the endogenous period later in life have been demonstrated in LL-reared pigmented mice, which showed a shorter period of activity rhythm compared to the DD-reared mice [29,47]. Since the endogenous period in mice is naturally shorter than 24 h, the LL rearing may just accentuate their “morning” chronotype. The endogenous period in rats is naturally longer than 24 h, so its prolongation after LL during development suggests the underscoring of their “evening” chronotype. In general, exposing adult nocturnal rodents to LL conditions reduces the amplitude of locomotor activity rhythms and lengthens the endogenous period, i.e., causes aftereffects [48]. It is possible that rearing in LL conditions causes long-term alterations in the speed of free-running rhythms, even when the animals experience 40 days of LD conditions. Mildly lower RA (Figure 1b) and IS (Figure 1c) values with significantly higher inter-individual variances between LL and LD groups may mean higher day-to-day variation and indicate a weaker pacemaking activity of the circadian clock in the LL-reared group of adult animals. 

In the next experiments, we tested the sensitivity to early-life LL conditions of the major circadian pacemaker in the SCN; the retina, which constitutes the light input pathway to the SCN; and the pineal gland, which is the important SCN output structure. It has been shown previously that a sex difference exists in the locomotor activity rhythm in LL occurring later in life in LL-reared rats [49]. We used four males and four females per time point in the P30 group and a combination of both sexes for the P90 group, with slightly more females. We compared the data between the sexes separately but did not find any difference between the males and females in any tissue or gene expression profile. Therefore, we present all the data together, and each time point represents males and females.

A major limitation of this study is the unequal temporal resolution between the P30 and P90 groups. While we collected the P30 tissue samples in 3 h intervals, we sampled only four time points at P90. This discrepancy does not allow for the precise comparison of cosinor parameters, such as phase, amplitude, and mesor between age groups. Therefore, we discuss the similarities and differences between the age groups only on the basis of two-way ANOVA comparison between LL and LD expression profiles and the assessment of rhythmicity loss or disclosure in the LL group.

In the SCN, all the examined genes displayed circadian rhythmicity in the control LD group, with the exception of *Bdnf*, which only emerged at P90 in the LL group. The enhancement of its rhythmicity in the LL group was also evident in the retina. Regarding its neuromodulatory role, such enhancement of its circadian amplitude suggests that *Bdnf* may potentially play a role in the mediating neuronal plasticity induced by non-physiological lighting conditions early in life. 

Ten days after transitioning from LL to LD, at P30, the expression profiles of *Per1, Per2, Nr1d1, Stat3*, and *Grin2B* in the SCN were significantly different from those of the controls. *Per1, Nr1d1, Stat3*, and *Grin2B* became arrhythmic in the LL group, and rhythms in *Per2* differed in mesor and amplitude. No change was measured by two-way ANOVA for *Bdnf* and *Grin1*, although their expression was also arrhythmic in the LL group. At P90, after 70 days of LD cycling, the rhythms of most of the genes were aligned with the controls, except the expression profile of *Grin2B*. Similar to P30, the expression profile of *Grin1* was arrhythmic in the LL group, but two-way ANOVA did not confirm the difference, possibly due to larger standard deviations. Our data resemble the previous observations that LL during lactation abolished rhythmicity in the vasoactive intestinal peptide, arginine vasopressin, and PER1 in the SCN, which was not restored by subsequent LD cycles until P90, indicating the permanent impairment of the SCN by LL during lactation [30]. Both subunits of NMDA receptors *Grin1* and *Grin2B* showed diurnal variations in the SCN also at the protein level, and *Grin2B* was involved in the light or glutamate-induced signaling cascades, leading to the clock gene expression in the SCN and subsequent phase shift of the clock [50,51,52]. We previously showed that *Grin2B* and *Grin1* expression in the hippocampus and cortex can be affected neonatally by the manipulation with GABAergic neurotransmission, suggesting their sensitivity to changes in the ionic milieu, which also varies in the SCN according to external lighting conditions [53,54]. The LL-induced alteration in *Grin2B* expression in the SCN can be a form of plasticity that may lead to an altered response of the circadian clock to light, which was observed in adult animals reared under LL [47].

Mammalian retina was the first tissue, outside of the SCN, described as displaying the circadian clock properties [55]. Since then, it has been demonstrated that the retina harbors a network of clocks localized in each cell layer. Bioluminescence recordings, using the luciferase reporter coupled to *Per1* and *Per2* clock genes, reported rhythmicity in ganglion cells, inner and outer nuclear layers, and photoreceptors [56,57,58]. Our analysis, however, did not prove the rhythmicity of these two core-clock genes in whole retinas of the control LD group at P30. Similarly to our data, Kamphuis et al. [59] showed blunted rhythms in the clock gene expression, especially in *Per1*, for whole extracted retinas. Their and our results may be related to the presence of a complex network of circadian clocks in various retinal layers, with differences in the phase and period between cells/layers that may mask the rhythmicity at the whole-tissue level. Hypothetically, better synchronization among retinal layers, or the strengthening of the clock in some layers, allows the clock gene rhythmicity to emerge at P90. An exception from this concept is *Nr1d1*, which showed rhythmicity in the LD group with a peak during the daytime already at P30. 

Blunted circadian rhythmicity was measured for three of four opsins in the retinas of P30 and P90 rats reared in LL. In rodent species, melanopsin mRNA rhythmic expression displays a peak in the transition between day and night [60,61,62,63], which corresponds with our results. The responsiveness of melanopsin to the LL condition has been tested in pigmented Brown Norwegian adult rats [63]. In contrast to our study, the prolonged LL conditions only changed the protein level but not the mRNA expression. However, the developmental aspects were not included in that study, and the ages of the animals and the light spectrum and intensity of constant light conditions were not specified. 

As reported previously, *Opn5* was observed in the rat retina [64], and in adult animals exposed to 8 days in LL, the levels of this opsin expression increased [65]. Our results show no circadian rhythmicity of *Opn5* expression, but a significant difference between groups. Similar to adult animals, the mean value of its expression was elevated in the LL-reared group compared to the LD groups. 

We also tested the changes in expression profiles of short-wavelength-sensitive cones. It is assumed that variations in visual opsins represent some adaptation to lighting habitat in various animal species and individuals [66]. However, our data show that *Opn1sw* did not change its expression in the P30 group or later in life. Concerning rhodopsin, its rhythmicity has been shown before [59]. We demonstrated its rhythmic expression in control animals at both ages, which was blunted by early development in LL. 

Acute light at night suppresses melatonin secretion from the pineal gland, which mostly results from the fast inhibition of AANAT transcription and activation [67,68]. However, the long-term effects of LL in adulthood or during development and plastic changes in the pineal circadian oscillations throughout life are unclear. In rodents, the pineal gland displays marked daily variations in the *Aanat* gene and clock gene expression [68,69,70,71]. Our data show robust circadian rhythmicity in the expression of all clock genes and both enzymes involved in the melatonin synthetic pathway and transcription factors *Stat3* and *Icer*. This rhythmicity was significantly blunted in animals that experienced LL conditions in early postnatal development. Although the rhythmicity persisted in the LL group, all profiles, except *Icer*, showed substantially lower mesor and amplitude at P30. This effect lasted until P90 for *Per1* and *Aanat*, a lower mesor was found in *Tph1*, and *Nr1d1* and *Stat3* expression become arrhythmic in the LL group at P90.

Together, all the genes in the pineal gland responded significantly to early life LL conditions, with the exception of the transcription factor *Icer*, whose expression profile did not change in any parameter in either group. The reason why the amplitude of the rhythm in *Icer* expression did not change, as it did in other genes, is not clear. The mammalian pineal gland is under strict control by the SCN, which regulates nocturnal increases in sympathetic noradrenaline and the subsequent β-adrenergic elevation of cAMP levels [72]. This pathway induces a marked increase in *Aanat* gene expression and the expression of clock genes *Per1, Per2*, and *Cry2*, but not *Bmal1* and *Nr1d1* [68,71]. After 36–60 h of LL, which disrupts noradrenergic SCN input, *Per1* and *Cry2* expression showed no rhythmicity in the pineal gland. However, the circadian rhythms in *Bmal1* and *Nr1d1* gene expression were unchanged and not altered, even by a β-adrenergic antagonist [71]. Similarly, the response of pineal *Per2* to light pulses at night was altered compared to *Per1* and *Aanat* [68]. The data suggest the existence of alternative or synergistic pathways that regulate circadian gene expression in the pineal gland. However, our data did not separate the clock genes in the pineal gland into two groups according to the response to LL conditions, which could be explained by distinct sensitivity to β-adrenergic signaling. Thus, although *Icer* should be affected by noradrenergic input as other genes [8,9,10,11], some other mechanism may allow it to resist external disruption by LL early in life. 

The exposure of pigmented rats to constant light from birth until P20 caused a decreased amplitude and mesor of seven of eight genes in the pineal gland at P30 and three of seven genes at P90, while *Stat3* became arrhythmic. In the SCN, five of the seven genes were arrhythmic in the LL group at P30, and *Grin2B* was shown arrhythmicity even at P90. In the retina, *Opn4* and *Rho* responded to postnatal LL conditions by the loss of rhythmicity and decreased amplitude in *Opn4* at P90, and *Opn5*, although non-rhythmic, showed a significantly different expression between groups. Interestingly, the expression profile of three core-clock genes in the LL group was either restored by P90 (in the SCN and, with the exception of *Per1*, also in the pineal gland) or, in the retina, not affected at all. This observation indicates that the other gene expression patterns perturbed at P90 may not be a direct consequence of disrupted circadian clock in every tissue. Glutamatergic neurotransmission and NMDA receptor composition undergo striking changes in the SCN early in postnatal development [50], which can be affected by altered chemical neurotransmission independently of circadian clockwork development [53,54]. The altered expression of opsins in the retina may indicate some adaptive photoreceptor arrangement upon early life lighting experiences, and the induction of long-term changes in the pineal gland may reflect altered β-adrenergic secretion in LL [73].

Altogether, our results indicate that postnatal light experience may affect temporal patterns in behavior and gene expression and suggest implications for the control of retinal physiology and pineal gland function later in life.

## Figures and Tables

**Table 1:** List of the TaqMan probes used in this study.

Gene Symbol	Ref. No.	Gene Name
*Aanat*	Rn01461110_m2	arylalkylamine *N*-acetyltransferase
*Bdnf*	Rn02531967_s1	brain-derived neurotrophic factor
*Gapdh*	Rn01775763_g1	glyceraldehyde 3-phosphate dehydrogenase
*Grin1*	Rn01436034_m1	glutamate receptor, ionotropic N-methyl D-aspartate 1
*Grin2b*	Rn00680474_m1	glutamate receptor, ionotropic N-methyl D-aspartate 2B
*Hprt*	Rn01527840_m1	hypoxanthine phosphoribosyltransferase 1
*Icer*	Rn04338541_m1	inducible cAMP early repressor
*Nr1d1*	Rn01460662_m1	Rev-Erbα
*Opn1sw*	Rn00578824_m1	opsin 1, short wavelength Sensitive
*Opn4*	Rn00593931_m1	melanopsin
*Opn5*	Rn00710997_m1	opsin 5
*Per1*	Rn01325256_m1	period 1
*Per2*	Rn01427704_m1	period 2
*Rho*	Rn00583728_m1	rhodopsin
*Stat3*	Rn00680715_m1	signal transducer and activator of transcription 3
*Tph1*	Rn01476867_m1	tryptophan hydroxylase 1

**Table 2:** Differences in cosinor parameters between control and LL-reared rats in SCN, retina, and the pineal gland, revealed by one-way ANOVA followed by Tukey’s multiple comparisons test. Significant values are in bold. Dashes indicate missing values. NA (not applicable) indicates that one or both profiles were not rhythmic and cosinor parameters could thus not be calculated (see Figure 2, Figure 3, Figure 4, Figure 5, Figure 6 and Figure 7 for details).

SCN		P30		P90		Retina		P30		P90		Pineal Gland		P30		P90	
		F=	*p* Value	F=	*p* Value			F=	*p* Value	F=	*p* Value			F=	*p* Value	F=	*p* Value
*Per1*	**mesor**	NA		**-**	-	*Per1*	mesor	NA		NA		*Per1*	mesor	12.240	**=0.0006**	5.230	**=0.0257**
	amplitude			-	-		amplitude						amplitude	11.560	**=0.0009**	4.521	**=0.0376**
	acrophase			-	-		acrophase						acrophase	0.305	=0.5816	0.656	=0.4210
*Per2*	mesor	12.840	**=0.0005**	0.573	=0.4522	*Per2*	mesor	NA		0.103	=0.7495	*Per2*	mesor	4.526	=0.0352	3.322	=0.0733
	amplitude	7.028	**=0.0091**	0.001	=0.9825		amplitude			0.189	=0.6655		amplitude	4.903	**=0.0285**	2.427	=0.1246
	acrophase	1.063	=0.3047	2.187	=0.1449		acrophase			9.218	**=0.0035**		acrophase	0.450	**=0.5033**	0.743	=0.3921
*Nr1d1*	mesor	NA		0.611	=0.4375	*Nr1d1*	mesor	NA		0.486	=0.4881	*Nr1d1*	mesor	16.250	**<0.0001**	0.699	=0.4066
	amplitude			0.282	=0.5973		amplitude			0.476	=0.4926		amplitude	42.960	**<0.0001**	0.021	=0.8846
	acrophase			1.007	=0.3198		acrophase			4.370	**=0.0406**		acrophase	2.807	=0.0962	1.772	=0.1881
*Bdnf*	mesor	NA		NA		*Bdnf*	mesor	NA		NA		*Bdnf*	mesor	6.881	**=0.0097**	-	-
	amplitude						amplitude						amplitude	4.087	**=0.0452**	-	-
	acrophase						acrophase						acrophase	7.194	**=0.0082**	-	-
*Stat3*	mesor	NA		3.265	=0.0762	*Stat3*	mesor	NA		-	-	*Stat3*	mesor	9.273	**=0.0028**	5.793	**=0.0193**
	amplitude			1.236	=0.2710		amplitude			-	-		amplitude	12.770	**=0.0005**	1.980	=0.1648
	acrophase			4.795	**=0.0328**		acrophase			-	-		acrophase	0.047	=0.8286	0.112	=0.7391
*Grin1*	mesor	NA		NA		*Aanat*	mesor	3.344	=0.0698	3.734	=0.0580	*Tph1*	mesor	9.218	**=0.0029**	3.562	**=0.0639**
	amplitude						amplitude	2.554	=0.1125	1.407	=0.2402		amplitude	10.290	**=0.0017**	1.157	=0.2864
	acrophase						acrophase	1.214	=0.2727	0.036	=0.8507		acrophase	0.407	=0.5244	4.460	**=0.0389**
*Grin2b*	mesor	NA		NA		*OPN4*	mesor	NA		4.445	**=0.0390**	*Aanat*	mesor	14.270	**=0.0002**	7.947	**=0.0065**
	amplitude						amplitude			0.025	=0.8759		amplitude	11.190	**=0.0011**	18.040	**<0.0001**
	acrophase						acrophase			5.149	**=0.0267**		acrophase	0.008	=0.9299	1.662	=0.2024
						*OPN5*	mesor	NA		NA		*Icer*	mesor	0.088	=0.7672	0.016	=0.8983
							amplitude						amplitude	0.506	=0.4782	0.005	=0.9461
							acrophase						acrophase	0.010	=0.9184	0.075	=0.7849
						*OPN1SW*	mesor	NA		NA							
							amplitude										
							acrophase										
						*Rho*	mesor	NA		NA							
							amplitude										
							acrophase										

## Supplementary Materials

The following are available online at https://www.mdpi.com/2227-9059/8/12/579/s1.
