# Supplementary material for: Constant Light in Critical Postnatal Days Affects Circadian Rhythms in Locomotion and Gene Expression in the Suprachiasmatic Nucleus, Retina, and Pineal Gland Later in Life
Source: Biomedicines. 2020 Dec 7;8(12):579. doi: 10.3390/biomedicines8120579 (PMC7762254; doi:10.3390/biomedicines8120579)
Supplement: Supplementary file 1 [file biomedicines-08-00579-s001.pdf]

Supplementary Information for: Constant light in critical postnatal days affects circadian rhythms in locomotion and gene expression in the suprachiasmatic nucleus, retina, and pineal gland later in life

Aneta Kubištová, Veronika Spišská, Lucie Petrželková, Leona Hrubcová, Simona Moravcová, Lenka Maierová and Zdeňka Bendová*

Department of Physiology, Faculty of Science, Charles University in Prague, Czech Republic

*Zdeňka Bendová (zdenka.bendova@natur.cuni.cz)

Contents:

Table 1: One-way ANOVA statistics to confirm the rhythmicity of gene expression in P30 and P90

Table 2: Cosinor analysis to confirm a significant circadian rhythmicity for all gene expression profiles

Table 3: Two-way ANOVA statistics revealed the difference between profiles of LL-reared and control animals

Table 1: One-way ANOVA statistics. One-way ANOVA was used to test the significance in variation in mRNA levels among nine daily time points.

**SCN**	**P30**				**P90**			
	controls		LL		controls		LL	
	F =	*p* value	F =	*p* value	F =	*p* value	F =	*p* value
Per1	5,848	<0.0001	2,385	= 0.0277	–	–	–	–
Per2	7,772	<0.0001	3,924	= 0.0009	17.790	<0.0001	5.758	= 0.0020
Nr1d1	4.033	= 0.0010	0.579	= 0.7909	3.920	= 0.0123	5.199	= 0.0031
Bdnf	0.172	= 0.9937	1.960	= 0.0705	1.685	= 0.1826	9.872	<0.0001
Stat3	3.028	= 0.0078	0.443	= 0.8898	5.784	= 0.0019	2.985	= 0.0374
Grin1	2.631	= 0.0173	0.708	= 0.6832	6.754	= 0.0007	1.531	= 0.2224
Grin2b	3.669	= 0.0019	0.859	= 0.5566	8.803	= 0.0001	0.489	= 0.7433

**Retina**	**P30**				**P90**			
	controls		LL		controls		LL	
	F =	*p* value	F =	*p* value	F =	*p* value	F =	*p* value
Per1	1.611	= 0.1419	0.667	= 0.7180	9.015	<0.0001	2.490	= 0.0643
Per2	1.708	= 0.1168	3.179	= 0.0056	10.370	<0.0001	6.929	= 0.0005
Nr1d1	2.184	= 0.0431	0.790	= 0.6137	8.740	<0.0001	3.940	= 0.0113
Bdnf	0.979	= 0.4625	3.432	= 0.0031	0.715	= 0.5884	3.855	= 0.0128
Stat3	0.828	= 0.5809	0.724	= 0.6694	–	–	–	–
Aanat	21.180	<0.0001	7.806	<0.0001	15.720	<0.0001	18.310	<0.0001
OPN4	4.842	= 0.0001	2.020	= 0.0602	12.500	<0.0001	17.280	<0.0001

OPN5	1.559	= 0.1570	1.723	= 0.1119	1.473	= 0.2369	1.792	= 0.1574
OPN1SW	1.039	= 0.4175	0.385	= 0.9243	5.448	= 0.0021	0.764	= 0.5577
Rho	5.851	<0.0001	0.564	= 0.8024	6.299	= 0.0008	5.539	= 0.8024

**Pineal gland**	**P30**				**P90**			
	controls		LL		controls		LL	
	F =	*p* value	F =	*p* value	F =	*p* value	F =	*p* value
Per1	28.880	<0.0001	8.804	<0.0001	17.37	<0.0001	10.910	<0.0001
Per2	13.810	<0.0001	7.373	<0.0001	8.357	= 0.0002	12.100	<0.0001
Nr1d1	49.080	<0.0001	9.902	<0.0001	3.523	= 0.0287	2.470	= 0.0667
Bdnf	7.598	<0.0001	8.899	<0.0001	–	–	–	–
Stat3	44.800	<0.0001	19.340	<0.0001	4.055	= 0.0115	1.841	= 0.1490
Tph1	21.620	<0.0001	6.048	<0.0001	6.756	= 0.0007	4.112	= 0.0093
Aanat	38.410	<0.0001	22.670	<0.0001	101.8	<0.0001	22.450	<0.0001
Icer	64.390	<0.0001	27.690	<0.0001	31.29	<0.0001	32.950	<0.0001

Table 2: Cosinor analysis. Cosinor analysis was used to confirm a significant circadian rhythmicity for all gene expression profiles

**SCN**	P30		P90	
	controls	LL	controls	LL
	p value		*p* value	
Per1	<0.0001	= 0.0677	–	–
Per2	<0.0001	<0.0001	<0.0001	= 0.0002
Nr1d1	<0.0001	= 0.3237	= 0.0013	= 0.0003
Bdnf	= 0.8617	= 0.0071	= 0.0798	<0.0001
Stat3	= 0.0001	= 0.4333	= 0.0070	= 0.0052
Grin1	= 0.0007	= 0.1378	<0.0001	= 0.3934
Grin2b	= 0.0002	= 0.1417	= 0.0007	= 0.3660

**Retina**	P30		P90	
	controls	LL	controls	LL
	p value		*p* value	
Per1	= 0.0069	= 0.5174	<0.0001	= 0.0186
Per2	= 0.0176	= 0.0176	<0.0001	<0.0001
Nr1d1	= 0.0108	= 0.4059	<0.0001	= 0.0025
Bdnf	= 0.0449	= 0.0007	= 0.6496	= 0.0053
Stat3	= 0.8226	= 0.1870	-	-
Aanat	<0.0001	<0.0001	<0.0001	<0.0001
Opn4	< 0,0001	= 0.0514	<0.0001	<0.0001

Opn5	= 0.2166	= 0.0357	= 0.2434	= 0.0339
Opn1sw	= 0.1336	= 0.4042	= 0.0013	= 0.2380
Rho	< 0,0001	= 0.5627	= 0.0293	= 0.0029

**Pineal gland**	P30		P90	
	controls	LL	controls	LL
	p value		*p* value	
Per1	<0.0001	<0.0001	<0.0001	<0.0001
Per2	<0.0001	<0.0001	<0.0001	<0.0001
Nr1d1	<0.0001	<0.0001	= 0.0001	= 0.0581
Bdnf	<0.0001	<0.0001	–	–
Stat3	<0.0001	<0.0001	= 0.0023	= 0.0702
Tph1	<0.0001	<0.0001	= 0.0002	= 0.0010
Aanat	<0.0001	<0.0001	<0.0001	<0.0001
Icer	<0.0001	<0.0001	<0.0001	<0.0001

Table 3: Two-way ANOVA statistics. Two-way ANOVA was used to analyse the difference between control and LL-reared animals in the SCN, retina and pineal gland.

**SCN**		P30		P90	
		F=	*p* value	F=	*p* value
Per1	interaction	3.179	= 0.0028	–	–
	time	6.583	<0.0001	–	–
	group	8.565	= 0.0042	–	–
Per2	interaction	2.367	= 0.0218	0.590	= 0.6716
	time	10.590	<0.0001	17.380	<0.0001
	group	10.400	= 0.0017	0.158	= 0.6929
Nr1d1	interaction	1.634	= 0.1240	0.305	= 0.8734
	time	2.857	= 0.0065	8.443	<0.0001
	group	8.459	= 0.0044	0.757	= 0.3880
Bdnf	interaction	1.007	= 0.4355	2.304	= 0.0698
	time	1.025	= 0.4221	6.855	= 0.0001
	group	0.062	= 0.8032	0.638	= 0.4280
Stat3	interaction	1.834	= 0.0791	2.367	= 0.0650
	time	2.929	= 0.0055	4.814	= 0.0023
	group	8.080	= 0.0054	1.771	= 0.1891
Grin1	interaction	1.521	= 0.1586	1.654	= 0.1744
	time	0.968	= 0.4648	6.089	= 0.0004
	group	0.640	= 0.4255	0.342	= 0.5610
Grin2b	interaction	3.119	= 0.0034	1.793	= 0.1433
	time	1.463	= 0.1798	3.292	= 0.0185

	group	7.113	= 0.0089	4.215	= 0.0448

Retina		P30		P90	
		F=	*p* value	F=	*p* value
Per1	interaction	0.553	= 0.9137	0.567	= 0.6874
	time	1.661	= 0.1160	9.565	<0.0001
	group	1.702	= 0.1947	0.767	= 0.3847
Per2	interaction	1.272	= 0.2663	2.281	= 0.0719
	time	3.590	= 0.0010	14.110	<0.0001
	group	0.002	= 0.9679	1.003	= 0.3209
Nr1d1	interaction	1.582	= 0.1385	1.388	= 0.2492
	time	1.369	= 0.2183	10.590	<0.0001
	group	0.004	= 0.9524	1.353	= 0.2494
Bdnf	interaction	1.811	= 0.0831	1.038	= 0.3955
	time	3.295	= 0.0021	3.426	= 0.0139
	group	2.593	= 0.1103	1.157	= 0.2866
Stat3	interaction	0.654	= 0.7304	–	–
	time	0.854	= 0.5572	–	–
	group	0.049	= 0.8254	–	–
Aanat	interaction	6.902	<0.0001	0.796	= 0.5328
	time	25.720	<0.0001	33.300	<0.0001
	group	4.614	= 0.0339	2.674	= 0.1076
Opn4	interaction	2.259	= 0.0278	1.883	= 0.1254
	time	4.971	<0.0001	26.900	<0.0001
	group	10.780	= 0.0014	6.866	= 0.0112
Opn5	interaction	1.326	= 0.2375	2.484	= 0.0537
	time	1.982	= 0.0545	0.606	= 0.6599
	group	6.557	= 0.0117	4.866	= 0.0314
Opn1sw	interaction	0.283	= 0.9705	1.328	= 0.2706
	time	1.052	= 0.4016	2.003	= 0.1064
	group	0.260	= 0.6112	0.085	= 0.7716
Rho	interaction	3.132	= 0.0030	1.340	= 0.2658
	time	2.273	= 0.0268	10.360	<0.0001
	group	4.499	= 0.0360	6.642	= 0.0125

Pineal gland		P30		P90	
		F=	*p* value	F=	*p* value
Per1	interaction	2,462	= 0.0166	2.612	= 0.3175
	time	33.550	<0.0001	29.020	= 0.0018
	group	15.350	= 0.0001	5.770	= 0.0198
Per2	interaction	1.583	= 0.1369	1.269	= 0.2932
	time	19.130	<0.0001	18.320	<0.0001
	group	5.816	= 0.0174	1.877	= 0.1761
Nr1d1	interaction	9.972	<0.0001	1.221	= 0.3124
	time	47.700	<0.0001	5.356	= 0.0010
	group	11.340	= 0.0010	1.379	= 0.2458

Bdnf	interaction	7.260	<0.0001	–	–
	time	8.976	<0.0001	–	–
	group	8.092	= 0.0052	–	–
Stat3	interaction	3.432	= 0.0014	1.209	= 0.3175
	time	59.580	<0.0001	4.934	= 0.0018
	group	11.340	= 0.0003	5.770	= 0.0198
Tph1	interaction	1.709	= 0.1031	1.887	= 0.1266
	time	23.420	<0.0001	9.839	<0.0001
	group	11.960	= 0.0008	4.829	= 0.0321
Aanat	interaction	4.504	<0.0001	13.240	<0.0001
	time	58.230	<0.0001	98.480	<0.0001
	group	20.820	<0.0001	10.910	= 0.0017
Icer	interaction	2.163	0.0349	0.268	= 0.8971
	time	79.910	<0.0001	62.970	<0.0001
	group	0.280	0.5976	0.176	= 0.6767
